# A systematic review of monitoring and evaluation indicators for sexual and reproductive health in humanitarian settings

**DOI:** 10.1186/s13031-019-0221-1

**Published:** 2019-10-14

**Authors:** Elena T. Broaddus-Shea, Loulou Kobeissi, Osama Ummer, Lale Say

**Affiliations:** 1 Horizons Global Health Consulting, Eagle, CO USA; 2 Department of Reproductive Health and Research, World Health Organization, Geneva, Switzerland; 3 Independent Consultant, New Delhi, India

**Keywords:** Sexual and reproductive health, Monitoring and evaluation, Humanitarian response, Conflict-affected settings

## Abstract

**Objective:**

To conduct a comprehensive mapping of published indicators for monitoring and evaluation (M&E) of sexual and reproductive health (SRH) services and outcomes in humanitarian settings.

**Methods:**

A systematic search of the peer-reviewed and grey literature published between January 2008 and May 2018 was conducted to identify all references describing indicator sets for M&E of SRH services and outcomes in humanitarian settings. The databases MEDLINE, Web of Science, and Global Health, as well as 85 websites of relevant organizations involved in humanitarian response were searched. Characteristics of identified indicator sets and data from individual indicators was extracted.

**Findings:**

Of **3278** records identified, **20** met the review’s inclusion criteria and **9** existing indicator sets were identified. A total of **179** relevant indicators were included in the mapping, and removal of duplicates yielded **132** unique indicators. Twenty-seven percent fell within the maternal health domain, followed by the HIV/AIDS domain (26%) and the gender-based violence domain (23%). The distribution of indicators by type (process/output, outcome, impact) was balanced overall but varied substantially across domains. The most commonly used data collection platforms were facility-based systems or population-based surveys. Domains covered and indicator definitions were inconsistent across indicator sets.

**Conclusion:**

Results demonstrate the need to standardize data collection efforts for M&E of SRH services and outcomes in humanitarian settings and to critically appraise the extent to which different domains should be covered. A core list of indicators is essential for assessing response status over time as well as across countries.

**Electronic supplementary material:**

The online version of this article (10.1186/s13031-019-0221-1) contains supplementary material, which is available to authorized users.

## Background

In line with target 3.7 of the Sustainable Development Goals (SDGs), access to sexual and reproductive health (SRH) services, including maternal health services, is crucial to ensure health and well-being of all people at all ages, and is a human right [1]. Yet ensuring access to SRH services is particularly challenging in humanitarian settings, given the collapse of health systems, limited quality of care and availability of human resources, as well as the increased vulnerabilities associated with conflict and displacement.

According to the Inter-agency Field Manual for Reproductive Health in Crisis, a humanitarian setting is “... one in which an event or series of events has resulted in a critical threat to the health, safety, security or well-being of a community or other large group of people. The coping capacity of the affected community is overwhelmed and external assistance is required. This can be the result of events such as armed conflicts, natural disasters, epidemics or famine, and often involves population displacement [2]”.

The Inter-Agency Working Group (IAWG) for reproductive health in crises provides guidance on six main objectives around the minimum initial service package (MISP) for reproductive health in crisis [2]. The MISP is a set of priority activities intended to be implemented immediately at the onset of crisis. The MISP also forms part of the Sphere Project’s minimum standards for humanitarian assistance [3]. Despite these established international standards for basic service provision in humanitarian settings, there remains no consensus around monitoring and evaluation (M&E) frameworks or sets of indicators to assess adequacy of SRH service provision in humanitarian settings as well their respective impacts on associated morbidity and mortality. Moreover, as time passes after the initial onset of an emergency and the setting passes into extended (or protracted) stages of crisis, service provision should move towards more comprehensive coverage of SRH needs [2]. Although M&E indicators and standards play an important role in guiding the transition to more comprehensive service provision, there are currently no widespread standards regarding core indicators that should be collected in extended stages in emergency settings versus those for acute stages.

Valid, timely, and reliable monitoring and evaluation data is essential for guiding effective humanitarian response as well as ensuring the accountability of all actors involved. Yet, often even the minimal needed data is unavailable [4]. Improving data availability and quality in humanitarian settings will require the commitment and willingness of the humanitarian actors across diverse agencies and organizations to invest in the time, effort and platforms to allow for the needed data to be collected. It will also require an openness for greater consistency in data collection, analysis, and use [4], in order to ensure comparability across settings and to demonstrate performance expectations for implementing organizations [5].

Given the need for increased focus on and consistency in the M&E of SRH services in humanitarian settings, the World Health Organization’s (WHO) Department of Reproductive Health and Research, in collaboration with the Department of Maternal, Child and Adolescent Health as well as numerous partner organizations and agencies, has committed to guide a collaborative and consultative review process. Ultimately, the goal is to propose a standardized set of core indicators for M&E of SRH services and outcomes in acute and extended humanitarian settings, and to provide guidance on their use. Initiated in April 2018 and expected to conclude in 2020, the review process consists of identifying current M&E indicators and mechanisms for SRH in humanitarian settings and convening in-depth stakeholder consultations to: assess their adequacy; standardize definitions and data collection procedures; and select and prioritize indicators for inclusion in a set of recommended indicators.

The process began with a systematic literature review conducted to identify current M&E indicators. An initial technical consultation which convened a wide variety of experts and other stakeholders was then held in December of 2018. The final step in the review process will involve field testing of standardized indicators and accompanying implementation recommendations in a variety of settings impacted by differing types and stages of humanitarian crises (April 2019–June 2020). Field testing will assess feasibility and allow for finalization of the core indicator sets across the different SRH domains, including establishing subsets specific to acute and extended stages of emergency.

## Main text

This paper seeks to describe the systematic literature review, which began this multi-year process and is intended to improve quality and consistency in the M&E of SRH services in humanitarian settings. This literature review served as the first step in the broader process and was conducted to describe and assess *existing* indicators published in the peer-reviewed and grey literature for SRH services and outcomes in humanitarian settings. Thus, it aimed to achieve the following objectives:

1. Identify existing indicator sets described within the peer-reviewed and grey literature, which are intended for the monitoring and evaluation of SRH services and outcomes in humanitarian settings.
2. Examine all relevant individual indicators within each set in order to assess the relative coverage of different SRH domains and topics, the relative frequency of indicator types (i.e. process, output, outcome, or impact), and to identify commonly occurring indicators.

## Methods

This review was conducted in accordance with the preferred reporting items for systematic review and meta-analysis protocols (PRISMA-P) guidance [6].

### Eligibility criteria

#### References published in the peer-reviewed literature, the grey-literature, and on websites were eligible for inclusion if they

- Described indicators for monitoring and/or evaluation of SRH in humanitarian settings
- Addressed multi-domain SRH services and outcomes
- Were published in English
- Were published after January 1st, 2008

#### References were excluded if any of the following criteria were relevant

- Not specific to humanitarian settings
- Not specific to SRH
- Addressed only a single SRH domain
- Did not describe specific indicators for monitoring or evaluating SRH services
- Described research other than monitoring and/or evaluation (i.e. needs assessments, retrospective analyses of DHS data)
- Described monitoring and/or evaluation of SRH-related interventions and services that were not health system-based (i.e. cash transfer program evaluations)

Humanitarian settings were defined according to the definition noted above from the Inter-agency Field Manual for Reproductive Health in Crisis. For the purposes of this review, SRH domains were defined in line with the MISP objectives from the Inter-Agency Field Manual on Reproductive Health in Humanitarian Settings, i.e.: Adolescent Reproductive Health (ARH), Family Planning (FP), Maternal Health (MH), Comprehensive Abortion Care (CAC), Gender-based Violence (GBV), Sexually Transmitted Infection (STI), and HIV/AIDS (HIV) [2]. The reason for including only references that addressed multiple (two or more) domains was due to the fact that even at the most minimal (such as the service package described in the MISP) SRH service provision in humanitarian settings must cover multiple domains. This inclusion criteria ensured that indicator sets identified in the review were those intended for assessing multi-domain SRH service packages, as opposed to siloed programs focused on a single domain. Date criteria were applied to ensure that materials retrieved reflected up-to-date practices and perspectives on monitoring and evaluation as well as of SRH.

### Information sources

Databases searched for peer-reviewed literature included: MEDLINE/PubMed, Web of Science, and Global Health. To identify grey-literature and online resources, a manual search was conducted of the websites of organizations that work extensively in humanitarian settings and/or do extensive work in the area of SRH.

### Search strategy

For the database search, search terms were selected by identifying relevant medical subject headings (MeSH) and keyword terms for the following concepts: sexual, reproductive, and maternal health; humanitarian settings; and M&E. The initial search was constructed in PubMed using “OR” to link terms for the same concept, and the term “AND” to link the groups of terms for different concepts. This was then translated into the correct syntax for the other two databases. Filters were applied to all searches to retrieve articles published in English since January 1st, 2008. The full search syntax for each database is available in the Additional file 1.

For the online search, an initial list of 60 organizations was compiled based on a list of participating agencies within the WHO Global Health Cluster. As potentially relevant web content and documents were identified while searching the websites of these organizations, the names of additional organizations mentioned (for example, collaborating partners on an initiative, or co-authors on a document) were recorded. The websites of these additional organizations were then searched as well. In total, 85 websites were searched (see Additional file 1 for complete list).

### Data management and selection process

Title, abstracts and other reference information for hits identified via the database search were downloaded to EndNote, and then exported in spreadsheet format. During the online search, all potentially relevant references were either downloaded as PDFs or saved as screenshots, and the bibliographic information for each (title, date, author, etc.) was entered into a spreadsheet. Two reviewers then independently screened the titles and abstracts of all peer-reviewed references and screened online references. Discrepancies in decisions about whether to include or exclude a particular reference were resolved through discussion. Next, the full-text of all references included during the initial round of screening were retrieved and reviewed. During this round of screening, reasons for exclusion were recorded and the list of references to include in the review was finalized.

### Data extraction & synthesis

First, metadata for indicator sets described was extracted from all references selected for inclusion during screening. This included: citation and name of indicator set, intended setting and stage of emergency, SRH domains examined, data sources used for indicators, and supporting resources available. Data for individual indicators were then extracted only for indicators that met the following criteria: 1) were specific to the health sector, 2) fell into one of the six SRH domains addressed by MISP objectives, and 3) could be defined in terms of specific, objective, and comparable numerators and denominators. These criteria were applied because the goal of this review was to identify indicators that would be comparable over time, across settings, and across emergency types. Finally, detailed information was extracted for each relevant indicator within the indicator sets identified. This included: source, domain, topic, name of indicator, definition, data source, and data collection method. Additionally, indicators were compared to those included in the monitoring and evaluation frameworks for the SDGs, the Global Strategy (GS) for Women’s Children’s and Adolescents’ Health, and WHO’s 100 Core Health Indicators [7–9]. Finally, indicators were classified by type (process/output, outcome, and impact), in line with the WHO Health Emergencies Program (work stream 4 on standardized indicator sets for acute and protracted event monitoring).

## Results

### Search results

**Fig. 1:**
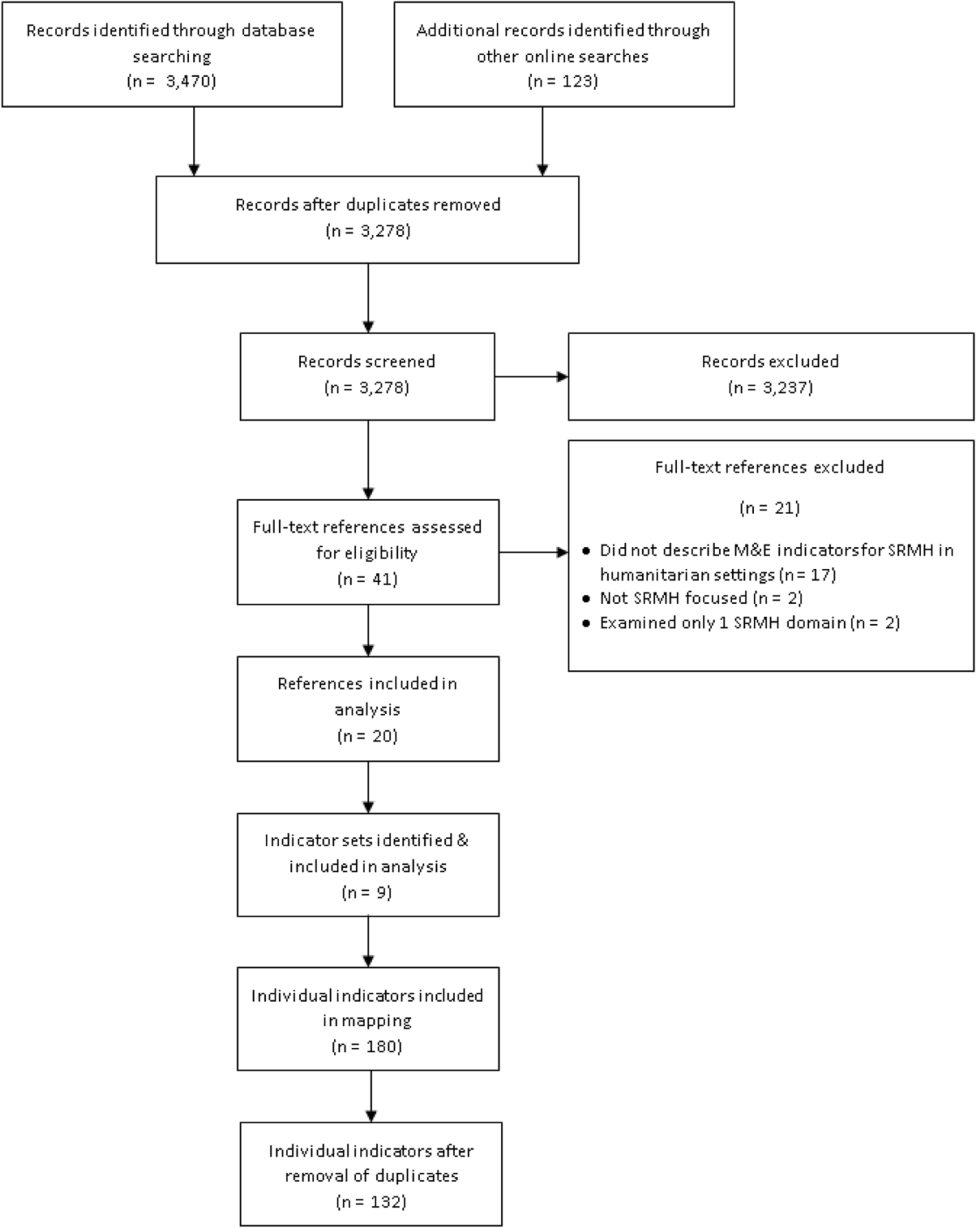
PRISMA Flowchart showing resource selection, indicator set identification, and indicator mapping process. ^1^ARH and CAC indicators not included since coverage of these domains is limited

As shown in Fig. 1, 3,470 records were retrieved from the database search, which resulted in 3155 unique hits after duplicates were removed. An additional 123 potentially relevant records were identified through online searching, yielding a total of **3278** records for screening. Of these, 3237 were excluded during the initial round of screening, and another 21 were excluded during full text screening. In total, **20** references were included in the analysis [3, 10–28]. From these 20 references, **9** existing indicator sets were identified. Finally, **179** relevant indicators from the indicator sets identified were included in the mapping. Removal of duplicates yielded **132** unique indicators.

Table 1 describes the 9 indicators sets that were identified from references included in the review (details for each reference are available in the Additional file 1). Table 2 provides the full list of unique indicators identified, organized by domain.

**Table 1:** Characteristics of indicator sets identified

Source, Year	Title	Intended Setting	Intended Stage of Emergency	Number Indicators Included in Mapping by SRMH Domain							Data Sources Used for Indicators					Resources Available
				ARH	CAC	FP	GBV	HIV	MH	STI	Facilities	Service Providers	Affected Population	Program Records	Not Specified	
IAWG, 2010 [15]	MISP Indicators	All humanitarian settings	Acute	0	0	0	2	3	1	0	**✓**				**✓**	• Some data collection guidance provided in IAFM manual
IAWG, 2010 [15]	Comprehensive Reproductive Health Service Indicators	All humanitarian settings	Extended	4	4	4	4	6	14	4	**✓**	**✓**	**✓**		**✓**	• Some data collection guidance provided in IAFM manual
CDC, 2013 [13]	Indicators for Pregnant and Postpartum Women after Disaster	Post-disaster settings in the United States	Acute	0	0	3	6	0	5	1			**✓**			• Extensive data collection guidance and data collection instruments available upon request^c^
	Health Information System Standards and Indicators	Camp and urban settings^a^	Both	1	0	2	4	22	19	4	**✓**					• Extensive data collection guidance, data collection instruments, data entry software, and automated analysis available online via UNHCR’s Twine system^d^ • System for ongoing data collection and reporting • Centralized data repository
CDC, 2007 [11]	Reproductive Health Assessment Toolkit for Conflict-Affected Women Key Indicator List	Conflict-affected settings	Both	0	0	6	13	7	8	3			**✓**			• Extensive data collection guidance, data collection instruments, and analysis guidance available online in Toolkit^e^ • Web-based trainings on data collection and data use also available online
Sphere Project, 2011 [3]	Sphere Standards Indicators	All humanitarian settings	Both	0	0	0	1	4	4	1	**✓**				**✓**	• Some guidance on data collection provided in Sphere Handbook
	Response Monitoring Indicator List for Maternal, Newborn and Child Health and Nutrition in Emergencies	All humanitarian settings	Both	0	0	0	0	2	3	0	**✓**	**✓**	**✓**	**✓**		• Some guidance on data collection provided in Guide to Maternal, Newborn and Child Health and Nutrition in Emergencies
	OCHA Indicators Registry	All humanitarian settings	Both	0	0	0	10	0	4	0	**✓**		**✓**		**✓**	• Guidance on data collection and suggested tools provided for some indicators but not others on registry website^f^ • Suggested data collection tools include the WHO’s SARA^g^ health facility assessment tool, and the HeRAMS^h^ approach
IAWG, 2017 [16]	MISP Process Evaluation Indicators^b^	All humanitarian settings	Both	–	–	–	–	–	–	–	**✓**	**✓**	**✓**			• Extensive data collection guidance, data collection instruments, and analysis guidance available online in Toolkit^i^

^a^Where health facilities are managed by UNHCR implementing partners-- two different versions available

^b^Not included in mapping as these indicators are intended for assessing MISP implementation, rather than monitoring SRMH services over time and across settings; however, this indicator set covers all domains

^c^This indicator set is part of the Reproductive Health Assessment after Disasters (RHAD) toolkit which is being updated and is projected to be available in 2020; data collection tools are currently available upon request from the CDC’s Emergency Preparedness and Response Team in the Division of Reproductive Health

^d^Available at: http://twine.unhcr.org/app/

^e^Available at: https://www.cdc.gov/reproductivehealth/global/tools/crisissituations.htm

^f^Available at: https://ir.hpc.tools/

^g^Service Availability and Readiness Assessment: available at http://www.who.int/healthinfo/systems/sara_introduction/en/

^h^Health Resources Availability Monitoring System: available at: http://www.who.int/hac/herams/en/

^i^Available at: http://iawg.net/resource/misp-process-evaluation-tools-2017/

**Table 2:** Unique indicators identified (*n* = 132)

Domain	Indicator (Sets it Appears In) [*italics* = appears in multiple sets]	Definition (Sets)	Type	Data Source					Overlap with Priority Indicators		
				Facilities	Service Providers	Affected Population	Program Records	Not Specified	SDG	GS	Core WHO
ARH	Condom use among young people (b)	Percentage of sexually active young people who reported using a condom at last intercourse	Outcome			✓					
ARH	Incidence of STDs in young people (b)	Number of reported cases of STDs among young people by the specified time period per 1000 young people	Impact	✓							
ARH	Proportion of births among those under 18 years (b)	Percentage of all live births which were deliveries among women under 18 years of age	Impact	✓					✓	✓	✓
ARH	*Proportion of STIs among those under 18 years (b, d)*	Percentage of total number of STIs diagnosed which were diagnosed among those under 18 years of age	Impact	✓							
CAC	Abortion services performed with appropriate technology (b)	Percentage of abortion services performed in a given period which are performed with appropriate technology (vacuum aspiration or medical methods)	Output	✓							
CAC	Awareness of legal indications for termination of pregnancy (b)	Percentage of providers involved in abortion services who are aware of the legal indications for termination of pregnancy	Output		✓						
CAC	Coverage of induced abortion (b)	Percentage of all women receiving abortion services in a given facility during a given period who receive induced procedures	Outcome	✓							
CAC	Coverage of post-abortion contraception (b)	Percentage of all women receiving abortion services in a given facility during a given period who receive modern contraceptive methods before leaving the facility	Outcome	✓							
FP	Barriers to family planning (e)	Percentage of women not currently using a family planning method who report at least one barrier to family planning (except for fertility-related reasons)	Output			✓					
FP	Difficulty accessing contraception after the disaster (c)	Proportion of PP women who have had difficulty accessing their contraceptive method since the disaster	Output			✓					
FP	Community knowledge concerning family planning (b)	Percentage of all sexually active persons targeted for family planning messages who are able to cite major messages about family planning	Output			✓					
FP	Ever heard of modern family planning methods (e)	Percentage of women of reproductive age who have ever heard of at least one modern family planning method	Output			✓					
FP	Contraceptive supply (b)	Percentage of service delivery points which maintain a minimum of 3 months’ supply of combined oral contraceptive pills, progestin-only pills, and injectables	Process	✓							
FP	Couple years protection (d)	Duration of contraceptive protection provided by all contraceptives sold or distributed free of charge to clients per 1-year period	Output	✓							
FP	Coverage of FP counselling (b)	Percentage of clients attending FP services who are offered FP counselling in addition to receiving a method of contraception	Output	✓							
FP	Unmet need for family planning (e)	Percentage of all women of reproductive age who are currently not using a family planning method and are at risk for pregnancy (not using a method, not currently pregnant or postpartum, fecund, sexually active in the last 30 days, and do not want a baby in the immediate future)	Outcome			✓					
FP	*Contraceptive prevalence (b, d, c)*	Percentage of women 15–49 years of age who are using (or whose partner is using) a contraceptive method (b, d); Proportion of PP women who are currently practicing family planning with their partner, including natural family planning methods (c)	Outcome	✓		✓					✓
FP	Modern contraceptive prevalence (e)	Percentage of all women of reproductive age who are using any modern family planning method	Outcome			✓					✓
FP	Use of family planning before disaster (c)	Proportion of PP women who were using a contraceptive method just before the disaster	Outcome			✓					
FP	Ever used modern family planning methods (e)	Percentage of women of reproductive age who have ever used at least one modern family planning method	Outcome			✓					
FP	Future intent to use a method in the next 12 months (e)	Percentage of women not currently using a family planning method who intend to use a family planning method in the next 12 months	Impact			✓					
GBV	*Availability of clinical management of rape survivors (a, h, f)*	Percentage of health facilities offering clinical management of rape survivors, including EC, PEP and presumptive STI treatment (a,h); Number of functional health facilities with clinical management of rape survivors in a defined administrative or health area at a certain time point (h); Not specified (f)	Output	✓						✓	
GBV	Percentage of health workers trained on clinical management of rape (h)	Percentage of all health workers which have been trained on Clinical Management of Rape	Output					✓			
GBV	Proportion of community-based workers trained in psychosocial support for GBV survivors (h)	Percentage of all community workers that have been trained in GBV psychosocial support	Output					✓			
GBV	Favorable to the continuation of FGC (e)	Percentage of women who have ever heard of FGC who think the practice should continue	Outcome			✓					
GBV	Future intent of FGC (e)	Percentage of women who have at least one daughter and have heard of FGC who intend to have youngest daughter’s genitals cut in the future	Outcome			✓					
GBV	Prevalence of female genital cutting (e)	Percentage of women of reproductive age who have ever had their genitals cut	Outcome			✓			✓	✓	
GBV	Prevalence of female genital cutting among youngest daughter that occurred in current setting (e)	Percentage of women who have at least one daughter and have heard of FGC whose youngest daughter ever had their genitals cut and the cutting was done in their current location	Outcome			✓					
GBV	*Number of reported rape cases (a, d)*	Number of rape cases reported to health facilities within a specific time period (time period for reporting to be set locally) (a); Number of rape cases reported per 10,000 population per year (d)	Impact	✓							
GBV	*Number of reported sexual violence cases (b, h)*	Number of cases of sexual violence reported to health services per month per 10,000 population	Impact	✓							
GBV	Communities indicating there is a risk of physical or sexual violence (h)	Percentage of all communities which indicate that there is a risk of physical or sexual violence	Outcome					✓			
GBV	Intimate partner violence (IPV) ever (e)	Percentage of ever-partnered women who have ever experienced IPV by a current or previous partner	Impact			✓					
GBV	IPV in past year (e)	Percentage of women partnered in the past 12 months who have experienced IPV in the past 12 months	Impact			✓			✓	✓	✓
GBV	Outsider physical violence during conflict (e)	Percentage of women of reproductive age who have experienced physical violence by someone outside of their family during the conflict	Impact			✓					
GBV	Outsider physical violence post-conflict (e)	Percentage of women of reproductive age who have experienced physical violence by someone outside of their family post-conflict	Impact			✓					
GBV	Outsider sexual violence during conflict (e)	Percentage of women of reproductive age who have experienced sexual violence by someone outside of their family during the conflict	Impact			✓					
GBV	Outsider sexual violence post-conflict (e)	Percentage of women of reproductive age who have experience sexual violence by someone outside of their family post-conflict	Impact			✓					
GBV	Physical intimate partner violence since disaster (c)	Percentage of pregnant women reporting physical violence by husband or partner since the disaster	Impact			✓					
GBV	Physical violence by family members in past year (e)	Percentage of women of reproductive age who have experienced physical violence by family members in the past year	Impact			✓					
GBV	Physical violence by persons other than intimate partners since disaster (c)	Percentage of pregnant women reporting physical violence by person other than husband or partner since the disaster	Impact			✓					
GBV	Sexual violence by anyone, including intimate partners since disaster (c)	Percentage of pregnant women reporting sexual violence by anyone including husband or partner since the disaster	Impact			✓					
GBV	Current needs for services for family violence (c)	Percentage of pregnant women reporting current need for services to reduce violence in family	Outcome			✓					
GBV	Perceived effect of violence on physical or emotional health (c)	Percentage of pregnant women reporting perceived effects of the violence on physical or emotional health	Impact			✓					
GBV	Sought treatment for effects of violence (c)	Percentage of pregnant women who have experienced violence since the disaster and sought treatment from a doctor, counsellor, or any other medical care provider for resulting physical and/or emotional problems	Impact			✓					
GBV	Humanitarian organizations and service providers with codes of conduct for own staff (h)	Percentage of humanitarian organizations and service providers that have in place codes of conduct on prevention of sexual exploitation and abuse by own staff	Output					✓			
GBV	Humanitarian organizations and service providers with community-based feedback and complaint mechanisms (h)	Percentage of humanitarian organizations and service providers that have in place community-based feedback and complaint mechanisms	Output					✓			
GBV	Reporting IPV (e)	Percentage of women who have ever experienced IPV who told an authority (doctor/provider, police, military, NGO worker) about any incidence of IPV	Impact			✓					
GBV	Reporting outsider violence (e)	Percentage of women who experienced outsider violence during and post-conflict who told an authority (doctor/provider, police, military, NGO worker) about any incident of outsider violence	Impact			✓					
GBV	*Timing of EC provision (b, d, h)*	Percentage of all rape cases reported within 120 h where survivors receive ECP within 120 h of incident (b, d); Percentage of female rape survivors who receive ECP within 120 h of the incident (h)	Outcome	✓							
GBV	*Timing of PEP provision (b, d, h)*	Percentage of all rape cases reported within 72 h where survivors receive PEP within 72 h of incident (b, d); Percentage of reported rape cases where survivor receives PEP for HIV within 72 h of incident (h)	Outcome	✓						✓	
GBV	*Timing of STI prophylaxis (b, d)*	Percentage of all rape cases reported in which survivors receive presumptive STI treatment within 2 weeks of an incident occurring (b); Percentage of all rape cases reported within 2 weeks where survivors receive presumptive STI treatment within 2 weeks (d)	Outcome	✓							
HIV	Eligibility for ART (d)	Number of people enrolled in HIV care and eligible for ART but not started on ART by end of period in one camp over a one-year time period	Impact	✓							
HIV	Number on ART (d)	Number of people started on ART in one camp over a one-month time period	Outcome	✓						✓	✓
HIV	Coverage of HIV rapid tests for safe blood transfusion (a)	Percentage of health service delivery points with sufficient HIV rapid tests to screen blood for transfusion	Output	✓							
HIV	*Quality of blood donation screening (b, d, f)*	Percentage of donated blood units screened for HIV in a quality assured manner (b, d); Not specified (f)	Outcome	✓							
HIV	*Condom distribution rate (d, a)*	Number of condoms distributed per person per month (d); Number of male condoms distributed per total population per month (a)	Output	✓							
HIV	*Condom use (b, e)*	Percentage of sexually active people reporting condom use at last intercourse (b); Percentage of women who had sex with a casual partner in the last 12 months who did not use a condom at last intercourse €	Outcome			✓					✓
HIV	Accepting attitudes of people living with HIV/AIDS (e)	Percentage of women who have ever heard of HIV/AIDS who indicate that they: do not believe HIV positive status of family member should be kept secret, are willing to care for HIV positive family member in home, believe HIV positive teacher should be allowed to continue teaching, and would buy fresh vegetables from an HIV positive person	Outcome			✓					
HIV	Comprehensive correct knowledge of HIV/AIDS (e)	Percentage of women of reproductive age who indicate that they: know condoms prevent HIV, know sex with only 1 faithful uninfected partner prevents HIV, do not think mosquitoes transmit HIV, do not think sharing food transmits HIV, and know a healthy-looking person can have HIV	Outcome			✓					
HIV	Comprehensive correct knowledge of mother-to-child transmission of HIV/AIDS (e)	Percentage of women of reproductive age who know that HIV/AIDS can be transmitted from mother to child during pregnancy or delivery, and through breastfeeding	Outcome			✓					
HIV	Perceived risk of getting HIV/AIDS (e)	Percentage of women who have ever heard of HIV/AIDS who believe they are at moderate to high risk of getting HIV/AIDS among women who have ever heard of HIV/AIDS	Outcome			✓					
HIV	Enrolled in HIV care who had TB status assessed (d)	Percentage of people enrolled in HIV care and seen for care who had TB status assessed and recorded during their last visit	Output	✓							
HIV	Targeting of people most at risk of exposure to HIV with a HIV prevention programme (f)	Not Specified	Output					✓			
HIV	Timing of PEP (f, g)	Percentage of individuals potentially exposed to HIV that receive PEP within 72 h of incident	Outcome				✓				
HIV	Infant HIV positive rate (d)	Percentage of all deliveries to HIV-positive mothers where the infant tested positive at 18 months of age	Impact	✓							
HIV	PMTCT ARV coverage (infant) (d)	Percentage of all deliveries to HIV-positive mothers where the infant swallowed ARV within 72 h of delivery	Outcome	✓							
HIV	PMTCT ARV coverage (mother) (d)	Percentage of all deliveries to HIV-positive mothers where the mother swallowed ARV during labour/delivery	Outcome	✓							✓
HIV	*PMTCT ARV coverage (mother-infant pair) (b, d)*	Percentage of all deliveries to HIV-positive mothers where the mother-infant pair swallowed ARV according to protocol	Outcome	✓							✓
HIV	PMTCT ARV use for treatment (d)	Percentage of all eligible HIV-infected pregnant women who received anti-retroviral as treatment	Outcome	✓							
HIV	Receipt of ARV drugs for PMTCT by pregnant women known to be HIV positive (f)	Not Specified	Outcome					✓			✓
HIV	PMTCT Co-trimoxazole prophylaxis (infant) (d)	Percentage of all deliveries to HIV-positive mothers where the infant was started on co-trimoxazole prophylaxis	Outcome	✓							
HIV	PMTCT Co-trimoxazole prophylaxis (mother) (d)	Percentage of all deliveries to HIV-positive mothers where the mother was started on co-trimoxazole prophylaxis	Outcome	✓							
HIV	*PMTCT pre-test counselling coverage (b, d)*	Percentage of first ANC visit clients who were pre-test counselled	Output	✓							
HIV	PMTCT Exclusive breastfeeding rate (d)	Percentage of all deliveries to HIV-positive mothers where the mother plans to exclusively breastfeed after delivery	Outcome	✓							
HIV	PMTCT Family planning acceptance rate (d)	Percentage of all deliveries to HIV-positive mothers where the mother accepted a modern method of family planning after delivery	Outcome	✓							
HIV	PMTCT HIV prevalence rate (d)	Percentage of all first antenatal care visits with HIV testing where the patient tested positive for HIV	Impact	✓							
HIV	PMTCT Home-based counselling (d)	Percentage of all deliveries to HIV-positive mothers where the mother received at least one home-based counselling visit after delivery	Output	✓							
HIV	PMTCT Post-test counselling and result (partners) (d)	Percentage of all antenatal care partners, who were pre-test counselled and HIV tested, who received post-test results and counselling	Output	✓							
HIV	*PMTCT post-test counselling and result (b, d)*	Percentage of first ANC visit clients tested for HIV who receive post-test result and counselling	Output	✓							
HIV	Proportional PMTCT service use by Nationals (d)	Percentage of all PMTCT clients pre-test counselled who were Nationals	Outcome	✓							
HIV	*Coverage of supplies for standard precautions (g, a)*	Percentage of health service delivery points with adequate supplies to carry out standard precautions	Output	✓							
HIV	Proportional VCT service use by nationals (d)	Percentage of all VCT clients pre-test counselled who are Nationals	Outcome	✓							
HIV	Received HIV test results in the last 12 months (e)	Percentage of women who were tested for HIV in the last 12 months who received their HIV test results	Output			✓					
HIV	*VCT post-test counselling and result (b, d)*	Percentage of VCT clients tested for HIV who received post-test result and counselling	Output	✓							
HIV	Would have an HIV test in the future (e)	Percentage of women who have ever heard of HIV/AIDS who would go for an HIV test in the future	Outcome			✓					
MH	*Complete ANC coverage (b, d, e)*	Percentage of total number of live births in which the mother made at least 4 ANC visits during the antenatal period at the time of delivery (b, d); Percentage of all women whose most recent pregnancy ended in a live birth or stillbirth in the last two years who received at least 3 antenatal care visits by a trained provider (e)	Outcome	✓		✓				✓	✓
MH	*Coverage of syphilis screening (b, d)*	Percentage of total number of live births where the mother had been screened for syphilis during the antenatal period at the time of delivery	Outcome	✓						✓	
MH	*Tetanus vaccination coverage (b, d)*	Percentage of total number of live births where the mother had received 2 doses of tetanus toxoid vaccine (or were fully vaccinated) during the antenatal period at the time of delivery	Outcome	✓							
MH	Timing of first antenatal visit (d)	Percentage of first-time antenatal visits that were made in the first trimester	Outcome	✓							
MH	Coverage of intermittent presumptive treatment for malaria (d)	Percentage of all live births where the mother had received two doses of fansidar prophylaxis during the antenatal period at the time of delivery	Outcome	✓							✓
MH	Coverage of long-lasting insecticidal nets (g)	Number of pregnant and lactating women and children under 5 sleeping nightly under net	Outcome				✓				
MH	*Percentage of caesarean section (b, d, f, h)*	Percentage of live births which are delivered via Caesarean sections	Outcome	✓							
MH	*Coverage of clean delivery kits (a, g, f)*	Number of clean delivery kits distributed per hundred pregnant women per month (a, g); Women in third trimester of pregnancy who have received clean delivery kits (f)	Output				✓				
MH	*Delivery assisted by a skilled attendant (b, d, h, e)*	Percentage of all deliveries which are attended by a trained health worker (b); Percentage of all live births attended by skilled birth attendants (d, h); Percentage of all women whose most recent pregnancy ended in a live birth or stillbirth in the last two years whose delivery was attended by a trained health care provider at a health facility (e)	Outcome	✓		✓			✓	✓	✓
MH	*BEmOC services availability (f, h)*	Number of functional health facilities with BEmOC per 500,000 population	Output	✓						✓	
MH	*CEmOC services availability (f, h)*	Number of functional health facilities with CEmOC per 500,000 population	Output	✓						✓	
MH	EmOC needs met (b)	Percentage of all deliveries with major obstetric complications which are treated at an EmOC facility	Outcome	✓							
MH	EmOC services availability (b)	Number of health facilities with basic and/or comprehensive obstetric care per 500,000 population by administrative unit	Output	✓						✓	
MH	*EmOC services utilization (b, d)*	Percentage of all deliveries which occur in an EmOC center	Outcome	✓							
MH	Health problems during pregnancy (c)	Percentage of women reporting health problems that require ongoing care. This includes diabetes, vaginal bleeding, urinary tract infections, sever nausea and vomiting, hypertensive disorders, heart problems, and any other identified by the interviewee.	Outcome			✓					
MH	Prevalence of anaemia (d)	Percentage of all antenatal mothers tested for anaemia with severe and moderate anaemia	Impact	✓							
MH	Prevalence of syphilis (d)	Percentage of antenatal mothers tested for syphilis that tested positive on Rapid Plasma Reagent testing	Impact	✓							
MH	Help-seeking behavior for postpartum complications (e)	Percentage of all women who reported postpartum complications after their most recent pregnancy that ended in a live birth or stillbirth in the last two years who sought help at a health facility	Outcome			✓					
MH	Help-seeking behavior for pregnancy complications (e)	Percentage of all women who reported complications before labor or delivery with their most recent pregnancy that ended in a live birth or stillbirth in the last two years who sought help at a health facility	Outcome			✓					
MH	Knowledge of danger signs of pregnancy complications (e)	Percentage of all women of reproductive age who know at least two danger signs of pregnancy complications	Output			✓					
MH	Pregnant women aware of where to go for labour and delivery (g)	Percentage of women who receive birth kits who receive counselling on the need for skilled birth attendance for delivery and know where to go	Output			✓					
MH	Proportion of low birth weight (c, b, d)	Proportion of postpartum women who reported their infants weighed less than 2500 g at birth (c); Percentage of total number of live births (with birth weight recorded) where the infant weighs less than 2500 g (b, d)	Impact	✓		✓					✓
MH	Preterm birth (c)	Proportion of PP women who reported they delivered a live singleton baby at least three weeks before their due date	Impact			✓					
MH	Direct obstetric case fatality rate (b)	Percentage of all women seen for a direct obstetric complication at an EmOC facility who die of a direct obstetric complication	Impact	✓							
MH	*Investigation of maternal deaths (b, d)*	Percentage of total number of reported maternal deaths which are investigated	Output	✓							✓
MH	Maternal mortality ratio (d)	Number of pregnancy-related deaths per 100,000 live births	Impact	✓					✓	✓	✓
MH	Number of maternal deaths reported (d)	Number of reported maternal deaths	Output	✓							
MH	Abortion ratio (d)	Number of abortions (defined as spontaneous miscarriage before 22 weeks gestation) per 1000 live births	Impact	✓							
MH	*Neonatal mortality rate (b, d)*	Number of live born infants who die at less than 28 days of age per 1000 live births in the specified period	Impact	✓					✓	✓	✓
MH	*Stillbirth rate (b, d)*	Number of still births per 1000 births (still and live) per month	Impact	✓						✓	✓
MH	Access to postpartum care (c)	Not specified	Output			✓					
MH	*Postnatal care coverage (b, d, e)*	Percentage of all live births where the mother received postnatal care 3 times within 6 weeks of delivery (b, d); Percentage of all women whose most recent pregnancy ended in a live birth or stillbirth in the last two years who received at least 1 postpartum care visit within six weeks after delivery (e)	Outcome	✓		✓				✓	✓
MH	Disaster related difficulty when accessing postpartum care (c)	Percentage of women who experienced difficulty obtaining a postpartum check-up because of the disaster	Outcome			✓					
MH	Crude birth rate (d)	Number of live births per 1000 total population	Impact	✓							
MH	Currently pregnant (e)	Percentage of all women of reproductive age who are currently pregnant	Impact			✓					
MH	Pregnancies in last two years (e)	Percentage of all women of reproductive age who have had one or more pregnancies in the last two years	Impact			✓					
STI	*Incidence of genital ulcer disease (b, d)*	Number of cases of genital ulcer disease per 1000 population per month	Impact	✓							✓
STI	*Incidence of male urethral discharge (b, d)*	Number of cases of male urethral discharge reported per 1000 population per month	Impact	✓							✓
STI	Selected STI-associated symptoms in the past 12 months (e)	Percentage of women of reproductive age who have had unusual genital discharge and/or genital ulcers or sores in the last 12 months	Impact			✓					✓
STI	Syphilis prevalence rate (OPD) (d)	Percentage of all STI patients tested who tested positive for syphilis using a pre-qualified syphilis test	Impact	✓							
STI	Help-seeking behaviors for treating selected STI-associated symptoms (e)	Percentage of women who had unusual genital discharge and/or genital ulcers or sores in the last 12 months who went to a health facility for treatment	Outcome			✓					
STI	Knowledge of selected STI-associated symptoms (e)	Percentage of women of reproductive age who know at least one of three selected STI-associated symptoms	Output			✓					
STI	Partner tracing (d)	Percentage of positive syphilis cases where contacts were tested	Output	✓							
STI	Access to STI services since disaster (c)	Not Specified	Output			✓					
STI	Primary healthcare facilities with antimicrobials to provide syndromic management to patients presenting with symptoms of an STI (f)	Not Specified	Output	✓							
STI	STI/RTI case management (b)	Percentage of total number of patients with STI/RTI accessing services that are assessed, treated and counselled according to protocol	Outcome	✓							
STI	STI/RTI management skills of service providers (b)	Percentage of service providers trained (or retrained) to manage STI/RTI cases according to protocol	Process		✓						

^1^Priority indicator sets include: The Sustainable Development Goal Monitoring Framework Indicators (SDG); the Global Strategy for Women, Children and Adolescents Monitoring and Evaluation Framework Indicators (GS); and the WHO’s Global Reference List of 100 Core Health Indicators (Core)

a: MISP Indicators

b: Comprehensive Reproductive Health Service Indicators

c: Indicators for Pregnant and Postpartum Women after Disaster

d: Health Information System Standards and Indicators

e: Reproductive Health Assessment Toolkit for Conflict-Affected Women Key Indicator List

f: Sphere Standards Indicators

g: Response Monitoring Indicator List for Maternal, Newborn and Child Health and Nutrition in Emergencies

h: OCHA Indicators Registry

### Coverage of SRH domains and topics within domains

As shown in Table 1, all indicator sets included indicators on MH, and all but one included indicators on GBV. Domains with the least coverage were those reporting on ARH and CAC. When looking at individual indicators, a similar trend emerged. The majority (27%) of the 132 unique indicators identified fell within the MH domain, followed by the HIV domain (26%) and the GBV domain (23%). Domains with the least coverage were ARH (3%) and CAC (3%).

**Fig. 2:**
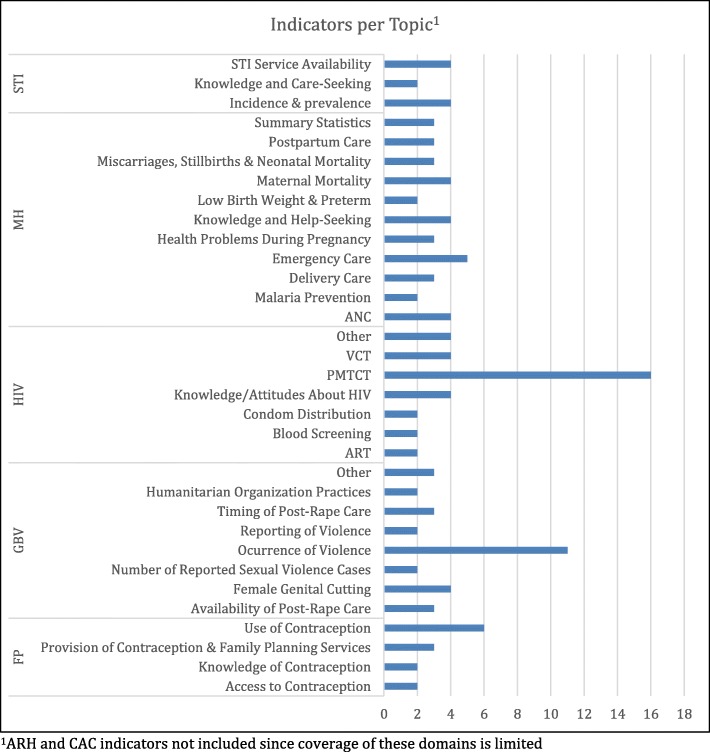
Coverage of topics within each SRMH domain across all unique indicators

For all domains other than ARH and CAC, indicators were also broken down by topic. Distributions by topic are shown in Fig. 2. Topics with the greatest coverage overall were prevention of mother-to-child transmission (PMTCT) (*n* = 16) within the HIV domain, and occurrence of violence (*n* = 11) within the GBV domain. Within other domains topics with the most coverage were STI service availability and STI incidence and prevalence (both *n* = 4), MH emergency care (*n* = 5), and use of contraception (*n* = 6).

The domain with the greatest breadth (number of different topics) was MH which had indicators covering 11 different topics. The number of indicators per topic was low, however, with topics covered by between 2 and 5 indicators. In contrast, the domains of GBV and HIV each included fewer topics (8 and 7, respectively) but had more indicators clustered within specific topics (occurrence of violence and PMTCT). The STI and FP domains had the fewest topics—3 and 4 respectively.

### Indicator types

**Fig. 3:**
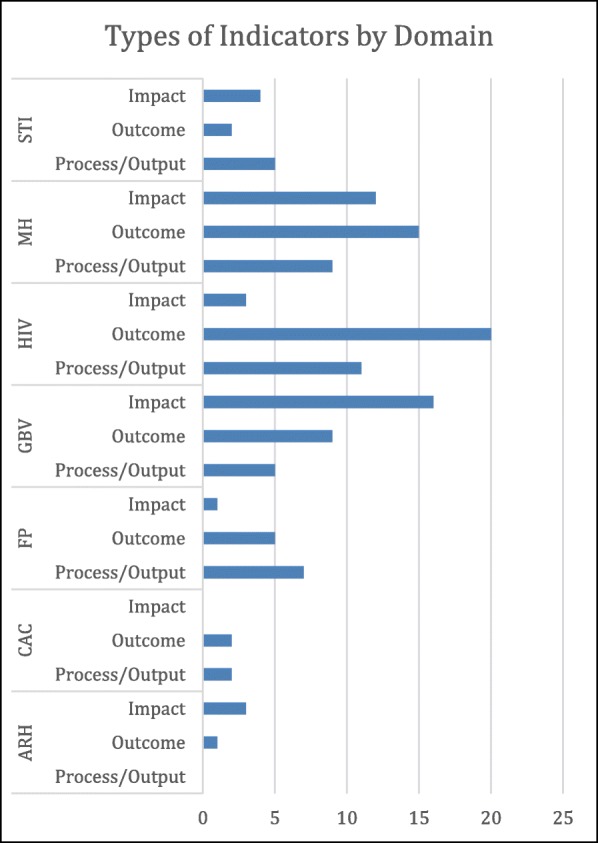
Indicator types by SRMH domain across all unique indicators

Overall the distribution of indicators by type (i.e. process/output, outcome, or impact) was fairly balanced, with the majority classified as outcome (41%), followed by impact (30%), and then by process/output (30%). When disaggregated by domain, as shown in Fig. 3, distributions of indicators by type varied substantially across domains. The greatest number of Impact indicators were in the GBV domain (*n* = 16), followed by the MH domain (*n* = 12). Numbers of outcome indicators were greatest in the HIV domain (*n* = 20) and in the MH domain (*n* = 15). These two domains also included the greatest number of process/output indicators (*n* = 11 and *n* = 9, respectively).

### Intended context for use

Of the 9 indicator sets identified, 6 were intended for use in all humanitarian settings, 1 was designed specifically for conflict-affected settings, 1 was designed for post-disaster settings in the United States, and 1 was intended for use with displaced populations in both camp and urban settings, with separate versions available for the two settings. Regarding stage of emergency, 6 indicator sets were intended for use during both acute and extended stages, 2 were intended specifically for the acute stage, and 1 was intended for extended or protracted stages. Interestingly, of the 6 indicator sets that indicated that they were appropriate for both acute and extended emergency stages, none specified which of the indicators included were appropriate during which stages.

### Data sources used

The majority of indicators (*n* = 65) used data only from facilities, meaning data obtained directly from facility records, entered into reporting systems by facility staff, or collected during facility assessments. Fifty indicators used data only from the affected population, obtained via population-based surveys. Five indicators could be calculated using data from either facilities or affected populations, depending on which definition was used for the indicator. For example, ‘complete antenatal care (ANC) coverage’ could be obtained using facility data when defined as, “percentage of total number of live births in which the mother made at least four ANC visits during the antenatal period at the time of delivery at facility,” but would require data from a population-based survey to calculate when defined as, “percentage of all women whose most recent pregnancy ended in a live birth or stillbirth in the last two years who received at least three ANC care visits by a trained provider.”

Aside from the indicators drawing on facility or population data, three indicators used data from program records. This includes, for example, the indicator on clean delivery kit coverage—this is intended to be calculated using data from the program distributing the kits on the total number distributed. Two indicators used data obtained directly from service providers regarding their knowledge and training. For seven indicators, it was unclear what data source should be used, and the set they were included in did not specify.

### Frequently occurring indicators & overlap with priority indicators

As shown in Tables 2 and 3, a total of 33 indicators appear in multiple sets. Of these, however, only 20 have definitions which are consistent across sets. As shown in Tables 2 and 4, 28 indicators overlapped with those included in the monitoring frameworks for the SDGs or the Global Strategy (GS), or in the WHO’s 100 Core Health Indicators (Core WHO). Less than half (only 11) had definitions which were consistent with the definition of the priority indicator.

**Table 3:** Indicators Appearing in Multiple Sets (*n* = 33)

Domain	Indicator	Number of Sets it Appears In^1^	Definitions Same Across Sets^1^
ARH	Proportion of STIs among those under 18 years	2	Yes
FP	Contraceptive prevalence	3	No
GBV	Number of reported rape cases	2	No
GBV	Number of reported sexual violence cases	2	Yes
GBV	Timing of EC provision	3	No
GBV	Timing of PEP provision	3	No
GBV	Timing of STI prophylaxis	2	No
GBV	Availability of clinical management of rape survivors	4	No
HIV	Quality of blood donation screening	3	No
HIV	Condom use	2	No
HIV	Timing of PEP	2	Yes
HIV	PMTCT ARV coverage (mother-infant pair)	2	Yes
HIV	Condom distribution rate	2	No
HIV	PMTCT pre-test counselling coverage	2	Yes
HIV	PMTCT post-test counselling and result	2	Yes
HIV	Coverage of supplies for standard precautions	2	Yes
HIV	VCT post-test counselling and result	2	Yes
MH	Proportion of low birth weight	3	No
MH	Neonatal mortality rate	2	Yes
MH	Stillbirth rate	2	Yes
MH	Complete ANC coverage	3	No
MH	Coverage of syphilis screening	2	Yes
MH	Tetanus vaccination coverage	2	Yes
MH	Percentage of caesarean section	4	Yes
MH	Delivery assisted by a skilled attendant	4	No
MH	EmOC services utilization	2	Yes
MH	Postnatal care coverage	3	No
MH	Coverage of clean delivery kits	3	No
MH	BEmOC services availability	2	Yes
MH	CEmOC services availability	2	Yes
MH	Investigation of maternal deaths	2	Yes
STI	Incidence of genital ulcer disease	2	Yes
STI	Incidence of male urethral discharge	2	Yes

**Table 4:** Indicators Overlapping with Those in Priority Indicator Sets (*n* = 28)

Domain	Indicator	Priority Indicator Sets with Overlap^a^	Definition Same as in Priority Indicator Set^b^
ARH	Proportion of births among those under 18 years	SDG, GS, Core	No
FP	Contraceptive prevalence	Core	No
FP	Modern contraceptive prevalence	Core	No
GBV	IPV in past year	SDG, GS, Core	Yes
GBV	Prevalence of female genital cutting	SDG, GS	No
GBV	Timing of PEP provision	GS	Yes
GBV	Availability of clinical management of rape survivors	GS	Yes
HIV	Number on ART	GS, Core	No
HIV	Condom use	Core	No
HIV	PMTCT ARV coverage (mother)	Core	No
HIV	PMTCT ARV coverage (mother-infant pair)	Core	No
HIV	Receipt of ARV drugs for PMTCT by pregnant women known to be HIV positive	Core	Yes
MH	Proportion of low birth weight	Core	Yes
MH	Maternal mortality ratio	SDG, GS, Core	Yes
MH	Neonatal mortality rate	SDG, GS, Core	Yes
MH	Stillbirth rate	GS, Core	Yes
MH	Complete ANC coverage	GS, Core	No
MH	Coverage of syphilis screening	GS	Yes
MH	Coverage of intermittent presumptive treatment for malaria	Core	No
MH	Delivery assisted by a skilled attendant	SDG, GS, Core	Yes
MH	Postnatal care coverage	GS, Core	No
MH	BEmOC services availability	GS	No
MH	CEmOC services availability	GS	No
MH	EmOC services availability	GS	Yes
MH	Investigation of maternal deaths	Core	No
STI	Incidence of genital ulcer disease	Core	No
STI	Incidence of male urethral discharge	Core	No
STI	Selected STI-associated symptoms in the past 12 months	Core	No

^a^Priority indicator sets include: The Sustainable Development Goal Monitoring Framework Indicators (SDG); the Global Strategy for Women, Children and Adolescents Monitoring and Evaluation Framework Indicators (GS); and the WHO’s Global Reference List of 100 Core Health Indicators (Core)

^b^When an indicator appeared in multiple sets and had multiple definitions, this question was marked yes if any of those definitions was the same as that in the priority indicator set

## Discussion

Findings from this review provided a comprehensive look at the existing indicators recommended for use for the purpose of M&E of SRH in humanitarian settings. Results clearly showed substantial variations across the different indicator sets in terms of the SRH domains covered, highlighted the different approaches taken towards data collection, and demonstrated discrepancies in indicator definitions across sets. The lack of consistency of coverage and definitions across indicator sets clearly indicates the need for greater harmonization. Differences in coverage observed across indicator domains may in part be due to changing emphases within the field of SRH over time. For example, both ARH and CAC are relatively newer areas of focus within SRH and are more politically challenging in comparison to other domains; this means there has been less time for and more challenges involved in developing indicators for these domains [15]. Regardless, these discrepant findings in the numbers and types of M&E indicators across the different SRH domains suggest the need for critically appraising the extent to which these domains should be covered during routine monitoring and evaluation, and whether development of additional indicators may be needed for adequate coverage for SRH in humanitarian settings.

Notable in their absence from the literature were indicator sets from many of the organizations that commonly implement relief efforts in emergency settings. Despite searching the websites of 85 organizations (many of which implement relief efforts), only one indicator set published by an implementing agency was identified [26]. This indicates that many organizations that provide SRH services in humanitarian settings do not make their M&E frameworks or indicator sets available in the public domain. Consequently, it is difficult to know which indicators are actually regularly used and reported on [4].

Broadly, our findings concur with the conclusions of Checchi et al. [4] in their review of public health information methods for crisis-affected populations. They assert the need for a common set of crisis-specific public health indicators, as well as establishment of a single health information platform for use in emergencies and a global data repository to store and analyze the data collected [4]. These needs underlie the consultative review process led by the WHO’s Department of Reproductive Health and Research (HRP) which aims to establish a recommended core set of SRH indicators for humanitarian settings.

Findings from this literature review have fed directly into the WHO’s consultative review process. The identified indicators, especially the 28 that overlapped with one or more of the priority indicator sets (i.e. either the SDG, GS or Core WHO indicators) (Table 4), served as a starting basis for the review process during a Technical Consultation with experts and stakeholder convened in December 2018. A report describing the progress of the consultative process, including results regarding indicator prioritization and standardization, were circulated to all partners who participated in the Technical Consultation and will be made available on the HRP website (https://www.who.int/reproductivehealth/publications/) upon incorporation of input from partners.

The prioritization process during the Technical Consultation revolved around selecting those indicators identified in this review which appeared in multiple sets and also overlapped with SDG, and/or GS, and/or WHO 100 Core Indicators. Along with prioritization, the review process focused on standardization—resolving inconsistencies across indicators sets and establishing clearly defined numerators, denominators, and data collection guidance for each indicator based on input from SRH experts and other stakeholders. Additionally, given the lack of indicator sets from implementing agencies identified in this review (as noted above), representatives from key implementing agencies participated in the Technical Consultation and contributed information about their internal M&E indicators and processes.

Ongoing areas of focus during the WHO’s consultative review process are indicator coverage and feasibility of usage. As this literature review demonstrates, coverage of existing indicators across domains varies substantially, and differs by indicator set. This raises questions regarding what the coverage of a core set of indicators *should* be, and what is most realistic. For example, this literature review identified few indicators in domains such as ARH and CAC—domains often associated with pertinent socio-political challenges that might prevent or hamper data collection. Another major feasibility question is not only whether the data collection for obtaining certain indicators would be logistically possible, but also whether it would be politically and bureaucratically feasible, making harmonization of indicators across settings difficult. Results from this literature review also indicate an uneven balance of indicators by data source, with the vast majority drawing on data from facilities or population-based surveys. Yet indicators drawing on other data sources, such as community-based indicators, may be more appropriate and informative for assessing services provided at levels beyond the health facility. Finally, for some SRH domains, such as HIV and GBV, crucial services are often provided by separate programs specific to these domains which are distinct from SRH services and programs. Therefore, ensuring appropriate coverage of the HIV and GBV domains within a core set of indicators will require multi-sector collaboration on the indicator selection process. These and other issues related to establishing a core SRH indicator set for humanitarian settings will continue to be explored during stakeholder consultations and via field-testing to assess indicator feasibility via collection of real-time data across varying humanitarian contexts.

In addition to the indicators identified, this review’s descriptions of the data collection tools, processes, and guidance that currently exist in association with each indicator set could be useful for identifying data collection platforms to scale up and harmonize data collection and reporting of indicators across agencies, settings, and time, as called for by Checchi et al. [4]. The extent to which supporting resources are available for data collection, analysis and reporting currently varies substantially across indicator sets. For instance, the indicator set identified to have the most extensive set of supporting resources is the UNHCR’s Health Information System Standards and Indicators, which is part of the Twine system (accessible at http://twine.unhcr.org/app/). The Twine system not only includes data collection tools and data entry templates, but also provides a mechanism for centralized reporting and automatic analysis. This is also the only set of indicators that is associated with an established system for ongoing data collection.

There is a need for greater emphasis on monitoring and evaluating SRH in humanitarian settings comprehensively, rather than taking a siloed approach. Only a few M&E studies examining a multi-domain set of SRH indicators were found in the peer-reviewed or grey-literature [14, 17, 22–25]. Instead, many studies focused on one single domain, such as MH or GBV, which hinders a general understanding of the status of SRH services and outcomes in humanitarian settings as a whole. The exception were those studies which examined the MISP implementation [14, 17, 23–25]. More specifically, the MISP Process Evaluation toolkit could be considered a valuable tool, given its broad coverage of multiple SRH domains across the six main MISP objectives. It should be noted, however, that although this toolkit is valuable, the data generated is focused on assessment of implementation processes, rather than M&E of SRH services and outcomes over time, or across settings.

Several strengths can be attributed to this review. These include its rigorous adherence to the PRISMA guidelines and the in-depth mapping process under-taken to synthesize key information from the 179 indicators identified. Additionally, focusing on the indicators themselves as the unit of analysis allowed for a unique and illuminating analysis. There are several limitations that should be equally noted. First, most of the indicator sets identified were either from guidance bodies (i.e. the Sphere Project, or the IAWG) or peer-review published literature, rather than directly reported from implementing agencies. As discussed above, this makes it difficult to accurately reflect the realities of M&E data collection efforts by the different implementing agencies from the field. Additionally, this review also does not indicate the feasibility and the practicality of collecting particular indicators in particular settings. Instead, feasibility will be assessed via field-testing at later stage in the WHO’s consultative review process. Finally, we only included English language studies. However, considering the global nature of this topic, we expect only very few eligible studies are missed by excluding non-English literature.

In addition to the consultative process currently underway, further research is needed to address these gaps, such as supplementing this information with field experience on what is being collected at the field level as well as seeking global consensus and a process of prioritization of a core list of M&E SRH indicators in humanitarian settings. Future studies should systematically examine the extent to which indicators are measuring what *should* be measured, vs. what *can* be measured, and which indicators and data collection methods are appropriate for use in which settings. Additionally, iterative participatory consultative processes, engaging a wide variety of stakeholders involved in humanitarian response—particularly those most connected to on-the-ground realities coupled with feasibility assessments—will be an essential component to culminate these efforts to standardize and harmonize indicators and to ensure scale up, accountability and commitment of partners to collecting some or all of the recommended M&E indicators.

## Conclusions

The results of this review assert the need for standardizing data collection efforts for M&E of SRH services and outcomes in humanitarian settings. A core list of indicators is essential for assessing response status over time as well as across and within countries. The 28 indicators identified via this review which overlap with either the SDGs, the Global Strategy or the 100 WHO Core indicators have provided the starting basis for an extensive consultative review process which aims to establish a standardized core indicator list. Rigorous reporting on a core list of indicators is a prerequisite for making the investment case that SRH response in humanitarian settings saves lives. Efforts are underway to conceptualize a core set of SRH indicators as well as to test their measurement feasibility. A standardized definition of accountability is a crucial bi-product of these efforts. A commitment by agencies on a core set of indicators requires a more conscious effort as well as willingness to share information and coordinate efforts. This could be possible by scaling up M&E of SRH efforts within the WHO’s global health cluster, as it could ensure measurement sustainability, especially so for protracted crises.

## Additional file

Additional file 1: Additional Supporting Resources and Guiding MESH terms. (DOCX 49 kb)

## Data Availability

Not applicable, however study protocol is registered with PROSPERO (CRD42018108492).

## Abbreviations

ARH: Adolescent reproductive health
CAC: Comprehensive abortion care
FP: Family planning
GBV: Gender-based violence
GS: GLOBAL Strategy for Women’s Children’s and Adolescents’ Health
HIV: Human immunodeficiency virus
IAWG: Inter-Agency Working Group
M&E: Monitoring and evaluation
MH: Maternal and newborn health
MISP: Minimum initial service package
PRISMA-P: Preferred reporting items for systematic review and meta-analysis protocols
SDGs: Sustainable Development Goals
SRH: Sexual and reproductive health
STI: Sexually transmitted infection
WHO: World Health Organization

## References

[1] Pillai V, Wang YC, Maleku A (2017). Women, war, and reproductive health in developing countries. Soc Work Health Care.

[2] Inter-agency Working Group on Reproductive Health in Crises (2010). Inter-agency field manual on reproductive health in humanitarian settings: 2010 revision for field review: Inter-agency Working Group on Reproductive Health in Crises.

[3] Sphere Project (2011). Sphere handbook: humanitarian charter and minimum standards in disaster response: the sphere Project.

[4] Checchi F, Warsame A, Treacy-Wong V, Polonsky J, van Ommeren M, Prudhon C (2017). public health information in crisis-affected populations: a review of methods and their use for advocacy and action. Lancet (London, England).

[5] Onyango MA, Hixson BL, McNally S (2013). Minimum initial service package (MISP) for reproductive health during emergencies: time for a new paradigm?. Glob Public Health.

[6] Moher D, Shamseer L, Clarke M, Ghersi D, Liberati A, Petticrew M (2015). Preferred reporting items for systematic review and meta-analysis protocols (PRISMA-P) 2015 statement. Systematic reviews.

[7] Leadership Council of the Sustainable Development Solutions Network. Indicators and a monitoring framework for the sustainable development goals: Launchng a data revolution for the SDGs. Sustainable Development Solutions Network, vol. 2015.

[8] Every Woman Every Child (2016). Indicator and Monitoring Framework for the Global Strategy for Women's Children's and Adolescents’ Health (2016–2030). Every Woman Every Child.

[9] World Health Organization (2015). Global reference list of 100 Core health indicators.

[10] CARE International (2017). Care emergency toolkit: sexual and reproductive health: CARE international.

[11] Centers for Disease Control and Prevention (CDC) (2007). Reproductive health assessment toolkit for conflict-affected women.

[12] Centers for Disease Control and Prevention (CDC) (2011). A process evaluation of the reproductive health assessment (RHA) toolkit for conflict-affected women: a report of findings, recommendations, and next steps.

[13] Centers for Disease Control and Prevention (CDC) (2013). Health indicators for disaster-affected pregnant and postpartum women and infants.

[14] Doedens W, Giga N, Krause S, Onyango MA, Sami S, Stone E, et al. Reproductive health Services for Syrian Refugees in Zaatri refugee camp and Irbid City, Jordan: an evaluation of the minimum initial service package: Boston University School of public health, United Nations Population Fund, US Centers for Disease Control and Prevention, Women's Refugee Commission; 2013.

[15] Inter-agency Working Group (IAWG) on Reproductive Health in Crises (2010). Inter-agency field manual on reproductive health in humanitarian settings.

[16] Inter-agency Working Group (IAWG) on Reproductive Health in Crises (2017). MISP Process Evaluation Tools.

[17] Krause S, Williams H, Onyango MA, Sami S, Doedens W, Giga N, Stone E, Tomczyk B (2015). Reproductive health services for Syrian refugees in Zaatri Camp and Irbid City, Hashemite Kingdom of Jordan: an evaluation of the Minimum Initial Services Package. Conflict Health.

[18] Moreland S, Curran J. A Guide for Monitoring and Evaluating Population- Health-Environment Programs, Second Edition. Chapel Hill: MEASURE Evaluation, University of North Carolina; 2018.

[19] Pyone T, Dickinson F, Kerr R, Boschi-Pinto C, Mathai M, van den Broek N (2015). Data collection tools for maternal and child health in humanitarian emergencies: a systematic review. Bull World Health Organ.

[20] UN OCHA. Indicator Registry 2016. Available from: https://ir.hpc.tools/. Accessed May 2019.

[21] UNHCR. Health information system (HIS) standards and indicators guide 2010. Available from: http://www.unhcr.org/protection/health/4614ab8e2/53-standards-indicators-guide-revised.html. Accessed May 2019.

[22] Whitmill J, Blanton C, Doraiswamy S, Cornier N, Schilperood M, Spiegel P, Tomczyk B (2016). Retrospective analysis of reproductive health indicators in the United Nations high commissioner for refugees post-emergency camps 2007-2013. Confl Health.

[23] Women's Commission for Refugee Women and Children. Reproductive health coordination gap, services ad hoc: minimum initial service package (MISP) assessment in Kenya: The Women's Commission for Refugee Women and Children, International Rescue Committee; 2008.

[24] Women's Refugee Commission. Evaluation of the MISP for reproductive health Services in Post-earthquake Nepal: Women’s Refugee Commission; 2016.

[25] Women's Refugee Commission, CARE International. International Planned Parenthood Federation, save the children. Priority reproductive health activities in Haiti: an inter-agency MISP assessment: Women's Refugee Commission; 2011.

[26] World Vision International. Guide to maternal, newborn and Child health and nutrition in emergencies: World Vision International; 2012.

[27] Zotti ME, Williams AM (2011). Reproductive health assessment after disaster: introduction to the RHAD toolkit. J Women's Health.

[28] Zotti ME, Williams AM, Wako E (2015). Post-disaster health indicators for pregnant and postpartum women and infants. Matern Child Health J.

