# Supplementary material for: A systematic review of monitoring and evaluation indicators for sexual and reproductive health in humanitarian settings
Source: Confl Health. 2019 Oct 14;13:43. doi: 10.1186/s13031-019-0221-1 (PMC6791013; doi:10.1186/s13031-019-0221-1)
Supplement: Supplementary file 1 — Additional Supporting Resources and Guiding MESH terms. (DOCX 49 kb) [file 13031_2019_221_MOESM1_ESM.docx]

## Resources identified via literature review and included in analysis

| **Author** | **Title** | **Publication Type** | **Reference Type** | **Indicator Sets Identified** |
| --- | --- | --- | --- | --- |
| Care International, 2017 | CARE Emergency Toolkit: Sexual & Reproductive Health section | Website | Guidance Manual | Sphere Standards Indicators |
| CDC, 2007 | Reproductive Health Assessment Toolkit for Conflict-Affected Women | Website | Data Collection Toolkit | Reproductive Health Assessment Toolkit for Conflict-Affected Women Key Indicator List |
| CDC, 2011 | A Process Evaluation of the Reproductive Health Assessment (RHA) Toolkit for Conflict-Affected Women: A report of findings, recommendations, and next steps | Grey-literature | Research Process Evaluation | Reproductive Health Assessment Toolkit for Conflict-Affected Women Key Indicator List |
| CDC, 2013 | Health Indicators for Disaster-Affected Pregnant and Postpartum Women and Infants | Grey-literature | Indicator List | Indicators for Pregnant and Postpartum Women after Disaster |
| Doedens et al., 2013 | Reproductive health services for Syrian refugees in Zaatri Camp and Irbid City, Hashemite Kingdom of Jordan: an evaluation of the Minimum Initial Services Package | Grey-literature | M&E Study | MISP Process Evaluation Indicator List |
| IAWG, 2010 | Inter-agency field manual on reproductive health in humanitarian settings: 2010 revision for field review | Grey-literature | Guidance Manual | MISP Indicators, Comprehensive Reproductive Health Services Indicators |
| IAWG, 2017 | MISP Process Evaluation Toolkit | Grey-literature | Data Collection Toolkit | MISP Process Evaluation Indicator List |
| Krause et al., 2015 | Reproductive health services for Syrian refugees in Zaatri Camp and Irbid City, Hashemite Kingdom of Jordan: an evaluation of the Minimum Initial Services Package | Peer-reviewed article | M&E Study | MISP Process Evaluation Indicator List |
| Measure Evaluation (date not specific) | Family Planning & Reproductive Health Indicators Database: Reproductive Health in Emergency Situations | Website | Indicator List | MISP Indicators |
| Pyone et al., 2015 | Data collection tools for maternal and child health in humanitarian emergencies: a systematic review | Peer-reviewed article | Literature Review | Health Information System Standards and Indicators, MISP Indicators, Comprehensive Reproductive Health Service Indicators, Reproductive Health Assessment Toolkit for Conflict-Affected Women Key Indicator List, Sphere Standards Indicators, Response Monitoring Indicator List for Maternal, Newborn and Child Health and Nutrition in Emergencies |
| The Sphere Project, 2011 | Sphere Handbook: Humanitarian Charter and Minimum Standards in Disaster Response, 2011 | Grey-literature | Guidance Manual | Sphere Standards Indicators |
| UN OCHA, 2016 | Indicators Registry | Website | Indicator List | OCHA Indicators Registry |
| UNHCR, 2014 | Health Information System (HIS) Standards and Indicators Guide | Website | Indicator List | Health Information System Standards and Indicators |
| Whitmill, et al., 2016 | Retrospective analysis of reproductive health indicators in the United Nations High Commissioner for Refugees post-emergency camps 2007-2013 | Peer-reviewed article | M&E Study | Comprehensive Reproductive Health Services Indicators |
| Women's Commission for Refugee Women and Children, 2008 | Reproductive Health Coordination Gap, Services Ad hoc: Minimum Initial Service Package (MISP) Assessment in Kenya | Grey-literature | M&E Study | MISP Process Evaluation Indicator List |
| Women's Refugee Commission, 2011 | Priority Reproductive Health Activities in Haiti: An inter-agency MISP assessment | Grey-literature | M&E Study | MISP Process Evaluation Indicator List |
| Women's Refugee Commission, 2016 | Evaluation of the MISP for Reproductive Health Services in Post-earthquake Nepal | Grey-literature | M&E Study | MISP Process Evaluation Indicator List |
| World Vision, 2016 | Guide to Maternal, Newborn and Child Health and Nutrition in Emergencies | Grey-literature | Guidance Manual | Response Monitoring Indicator List for Maternal, Newborn and Child Health and Nutrition in Emergencies |
| Zotti & Williams, 2011 | Reproductive Health Assessment After Disaster: Introduction to the RHAD Toolkit | Peer-reviewed article | Data Collection Toolkit | Indicators for Pregnant and Postpartum Women after Disaster |
| Zotti et al., 2015 | Post-disaster Health Indicators for Pregnant and Postpartum Women and Infants | Peer-reviewed article | Indicator List | Indicators for Pregnant and Postpartum Women after Disaster |

## Search terms

|  | PubMed Syntax | Web of Science Syntax | Global Health Syntax |
| --- | --- | --- | --- |
| Sexual, reproductive, and maternal health | "Reproductive Health Services"[Mesh] OR "Reproductive Health"[Mesh] OR “Reproductive Medicine” [Mesh] OR “reproductive health” [TIAB] OR “Maternal Health”[Mesh] OR “maternal health” [TIAB] OR "Sexual Health"[Mesh] OR “sexual health”[TIAB] OR "Maternal Mortality"[Mesh] OR “maternal mortality”[TIAB] OR “maternal morbidity”[TIAB] OR "Contraception"[Mesh] OR "Contraception Behavior"[Mesh] OR “Contraceptive Devices”[Mesh] OR “Contraceptive Agents, Female”[Mesh] OR “contraception”[TIAB] OR “contraceptive”[TIAB] OR “condom”[TIAB] OR “condoms”[TIAB] OR “family planning”[TIAB] OR “birth control”[TIAB] OR “birth spacing”[TIAB] OR “the pill”[TIAB] OR “microbicide”[TIAB] OR “microbicides”[TIAB] OR “diaphragm”[TIAB] OR “diaphragms”[TIAB] OR “IUD”[TIAB] OR “intrauterine device”[TIAB] OR “intrauterine devices”[TIAB] OR “implant”[TIAB] OR “implants”[TIAB] OR “Obstetric Surgical Procedures”[Mesh] OR “Pregnancy Complications”[Mesh] OR “abortion”[TIAB] OR “abortions”[TIAB] OR “abortal”[TIAB] OR “abortals”[TIAB] OR “postabortion”[TIAB] OR “post-abortion”[TIAB] OR "Sexually Transmitted Diseases"[Mesh] OR “sexually transmitted disease”[TIAB] OR “sexually transmitted infection”[TIAB] OR “sexually transmitted diseases”[TIAB] OR “sexually transmitted infections”[TIAB] OR "HIV Infections"[Mesh] OR “HIV”[Mesh] OR “Human Immunodeficiency Virus”[TIAB] OR “Acquired Immunodeficiency Syndrome”[TIAB] OR “HIV”[TIAB] OR “HIV/AIDS”[TIAB] OR “AIDS”[TIAB] OR “mother-to-child transmission”[TIAB] OR “maternal transmission”[TIAB] OR “MTCT”[TIAB] OR “PMTCT”[TIAB] OR "Pregnancy Outcome"[Mesh] OR “pregnancy”[TIAB] OR “pregnant”[TIAB] OR “prenatal care” [TIAB] OR “antenatal care” [TIAB] OR “emergency obstetric care” [TIAB] OR “safe pregnancy” [TIAB] OR “safe motherhood” [TIAB] OR “minimum initial service package” [TIAB] OR “Domestic Violence”[Mesh] OR “Gender-Based Violence”[Mesh] OR “Intimate Partner Violence”[Mesh] OR “Sex Offenses”[Mesh] OR “sexual harassment”[Mesh] OR “Battered Women”[Mesh] OR “domestic violence”[TIAB] OR “gender-based violence”[TIAB] OR “partner violence”[TIAB] OR “family violence”[TIAB] OR “violence against women”[TIAB] OR “partner abuse”[TIAB] OR “spousal abuse”[TIAB] OR “spouse abuse”[TIAB] OR “wife abuse”[TIAB] OR “sexual abuse”[TIAB] OR “sex crime”[TIAB] OR “sexual crime”[TIAB] OR “sexual violence”[TIAB] OR “rape”[TIAB] OR “sexual assault”[TIAB] OR “sexual harassment”[TIAB] OR “sexual coercion”[TIAB] OR “forced sex”[TIAB] OR “sexual slavery”[TIAB] OR “abused woman”[TIAB] OR “abused women”[TIAB] OR “battered woman”[TIAB] OR “battered women”[TIAB] | TS=(“reproductive health” OR “maternal health” OR “sexual health” OR “maternal mortality” OR “maternal morbidity” OR “contraception” OR “contraceptive” OR “condom” OR “condoms” OR “family planning” OR “birth control” OR “birth spacing” OR “the pill” OR “microbicide” OR “microbicides” OR “diaphragm” OR “diaphragms” OR “IUD” OR “intrauterine device” OR “intrauterine devices” OR “implant” OR “implants” OR “abortion” OR “abortions” OR “abortal” OR “abortals” OR “postabortion” OR “post-abortion” OR “sexually transmitted disease” OR “sexually transmitted infection” OR “sexually transmitted diseases” OR “sexually transmitted infections” OR “Human Immunodeficiency Virus” OR “Acquired Immunodeficiency Syndrome” OR “HIV” OR “HIV/AIDS” OR “AIDS” OR “mother-to-child transmission” OR “maternal transmission” OR “MTCT” OR “PMTCT” OR “pregnancy” OR “pregnant” OR “prenatal care” OR “antenatal care” OR “emergency obstetric care” OR “safe pregnancy” OR “safe motherhood” OR “minimum initial service package” OR “domestic violence” OR “gender-based violence” OR “partner violence” OR “family violence” OR “violence against women” OR “partner abuse” OR “spousal abuse” OR “spouse abuse” OR “wife abuse” OR “sexual abuse” OR “sex crime” OR “sexual crime” OR “sexual violence” OR “rape” OR “sexual assault” OR “sexual harassment” OR “sexual coercion” OR “forced sex” OR “sexual slavery” OR “abused woman” OR “abused women” OR “battered woman” OR “battered women”) | “reproductive health” OR “maternal health” OR “sexual health” OR “maternal mortality” OR “maternal morbidity” OR “contraception” OR “contraceptive” OR “condom” OR “condoms” OR “family planning” OR “birth control” OR “birth spacing” OR “the pill” OR “microbicide” OR “microbicides” OR “diaphragm” OR “diaphragms” OR “IUD” OR “intrauterine device” OR “intrauterine devices” OR “implant” OR “implants” OR “abortion” OR “abortions” OR “abortal” OR “abortals” OR “postabortion” OR “post-abortion” OR “sexually transmitted disease” OR “sexually transmitted infection” OR “sexually transmitted diseases” OR “sexually transmitted infections” OR “Human Immunodeficiency Virus” OR “Acquired Immunodeficiency Syndrome” OR “HIV” OR “HIV/AIDS” OR “AIDS” OR “mother-to-child transmission” OR “maternal transmission” OR “MTCT” OR “PMTCT” OR “pregnancy” OR “pregnant” OR “prenatal care” OR “antenatal care” OR “emergency obstetric care” OR “safe pregnancy” OR “safe motherhood” OR “minimum initial service package” OR “domestic violence” OR “gender-based violence” OR “partner violence” OR “family violence” OR “violence against women” OR “partner abuse” OR “spousal abuse” OR “spouse abuse” OR “wife abuse” OR “sexual abuse” OR “sex crime” OR “sexual crime” OR “sexual violence” OR “rape” OR “sexual assault” OR “sexual harassment” OR “sexual coercion” OR “forced sex” OR “sexual slavery” OR “abused woman” OR “abused women” OR “battered woman” OR “battered women” |
| Humanitarian settings | "Refugees"[Mesh] OR "Refugees" [TIAB] OR "Warfare"[Mesh] OR "war"[TIAB] OR “wars”[TIAB] OR "Armed Conflicts"[Mesh] OR "armed conflict"[TIAB] OR “armed conflicts”[TIAB] OR "Ethnic Violence"[Mesh] OR "ethnic violence"[TIAB] OR "ethnic conflict"[TIAB] OR "ethnic conflicts"[TIAB] OR "Disasters"[Mesh] OR "disaster"[TIAB] Or "disasters"[TIAB] OR "Relief Work"[Mesh] OR "relief work"[TIAB] OR "Disease Outbreaks"[Mesh] OR "disease outbreak"[TIAB] OR "disease outbreaks"[TIAB] OR "protracted crisis"[TIAB] OR "forced migration"[TIAB] OR "forced displacement"[TIAB] OR "internally displaced"[TIAB] OR "humanitarian"[TIAB] OR "fragile state"[TIAB] OR "fragile states"[TIAB]OR "conflict affected"[TIAB] OR "asylum"[TIAB] OR "complex emergency"[TIAB] OR “complex emergencies”[TIAB] | TS=("Refugees" OR "war" OR “wars” OR "armed conflict" OR “armed conflicts” OR "ethnic violence" OR "ethnic conflict" OR "ethnic conflicts" OR "disaster" Or "disasters" OR "relief work" OR "disease outbreak" OR "disease outbreaks" OR "protracted crisis" OR "forced migration" OR "forced displacement" OR "internally displaced" OR "humanitarian" OR "fragile state" OR "fragile states” OR "conflict affected" OR "asylum" OR "complex emergency" OR “complex emergencies”) | "Refugees" OR "war" OR “wars” OR "armed conflict" OR “armed conflicts” OR "ethnic violence" OR "ethnic conflict" OR "ethnic conflicts" OR "disaster" Or "disasters" OR "relief work" OR "disease outbreak" OR "disease outbreaks" OR "protracted crisis" OR "forced migration" OR "forced displacement" OR "internally displaced" OR "humanitarian" OR "fragile state" OR "fragile states “OR "conflict affected" OR "asylum" OR "complex emergency" OR “complex emergencies” |
| Monitoring & evaluation | "Public Health Systems Research"[Mesh] OR "Quality Assurance, Health Care"[Mesh] OR "Health Care Evaluation Mechanisms"[Mesh] OR “monitor”[TIAB] OR “monitoring”[TIAB] OR “evaluate”[TIAB] OR “evaluating”[TIAB] OR “evaluation”[TIAB] OR “evaluations”[TIAB] OR “assessment”[TIAB] OR “assessments”[TIAB] OR “framework”[TIAB] OR “frameworks”[TIAB] OR “indicator”[TIAB] OR “indicators”[TIAB] OR “data collection“[TIAB] OR “accountability”[TIAB] OR “effectiveness”[TIAB] OR “surveillance”[TIAB] | “monitor” OR “monitoring” OR “evaluate” OR “evaluating” OR “evaluation” OR “evaluations” OR “assessment” OR “assessments” OR “framework” OR “frameworks” OR “indicator” OR “indicators” OR “data collection“ OR “accountability” OR “effectiveness” OR “surveillance” | “monitor” OR “monitoring” OR “evaluate” OR “evaluating” OR “evaluation” OR “evaluations” OR “assessment” OR “assessments” OR “framework” OR “frameworks” OR “indicator” OR “indicators” OR “data collection“ OR “accountability” OR “effectiveness” OR “surveillance” |

## List of Websites Searched

| **Organization** | **URL** |
| --- | --- |
| African Population and Health Research Center | <http://aphrc.org/> |
| Aga Khan Development Network | <http://www.akdn.org/> |
| Australian Aid | <http://dfat.gov.au/aid/Pages/australias-aid-program.aspx> |
| Belgian First Aid & Support Team (B-Fast) | <https://b-fast.be/en> |
| Bill and Melinda Gates Foundation | <https://www.gatesfoundation.org/> |
| Bureau of Population, Refugees, and Migration/Dept of State | <https://www.state.gov/j/prm/> |
| Cambridge Reproductive Health Consultants | <http://www.cambridgereproductivehealthconsultants.org/> |
| Canada Global Affairs, International Humanitarian Assistance | <http://international.gc.ca/world-monde/issues_development-enjeux_developpement/response_conflict-reponse_conflits/humanitarian_assistance-aide_humanitaire.aspx?lang=eng> |
| CARE International | <https://www.care-international.org/> |
| Center for Global Development | <https://www.cgdev.org/> |
| Center for Health and Gender Equity | <http://www.genderhealth.org/> |
| Center for Humanitarian Health | <http://www.hopkinshumanitarianhealth.org/> |
| Center for Reproductive Rights | <https://www.reproductiverights.org/> |
| Centers for Disease Control and Prevention | <https://www.cdc.gov/index.htm> |
| Community Partners International | <https://hhi.harvard.edu/publications/data-preparedness-connecting-data-decision-making-and-humanitarian-response> |
| Consortium of Reproductive Health Association (CORHA) | <http://corhaethiopia.org/> |
| Cordaid | <https://www.cordaid.org/en/> |
| CPC Learning Network | <http://www.cpcnetwork.org/> |
| Danish Ministry of Foreign Affairs | <http://um.dk/en/> |
| DFID | <https://www.gov.uk/government/organisations/department-for-international-development> |
| Eastern Mediterranean Public Health Network | <http://emphnet.net/> |
| Elrha | <http://www.elrha.org/> |
| Emory University, Center for Humanitarian Emergencies | <http://che.emory.edu/> |
| Engender Health | <https://www.engenderhealth.org/> |
| Family Planning 2020 | <http://www.familyplanning2020.org/> |
| GBV Prevention Network | <http://preventgbvafrica.org/> |
| Gender-based Violence Area of Responsibility Working Group (GBV AoR) | <https://gbvguidelines.org/en/home/> |
| GIZ | <https://www.giz.de/en/html/index.html> |
| Global Women's Institute | <https://globalwomensinstitute.gwu.edu/> |
| Guttmacher Institute | <https://www.guttmacher.org/> |
| Gynuity Health Projects | <http://gynuity.org/> |
| Harvard Humanitarian Initiative | <https://hhi.harvard.edu/> |
| Healthy Newborn Network | <https://www.healthynewbornnetwork.org/> |
| Human Rights Watch | <https://www.hrw.org/> |
| Humanitarian Response | <https://www.humanitarianresponse.info/> |
| Humanity First | <https://uk.humanityfirst.org/> |
| Humedica International Hilfe | <https://www.humedica.org/index_eng.html> |
| Institute for Reproductive Health- Georgetown University | <http://irh.org/> |
| Interagency Standing Committee (IASC) | <https://interagencystandingcommittee.org/> |
| Inter-Agency Working Group on Reproductive Health in Crises | <http://iawg.net/> |
| Internal Displacement Monitoring Centre (IDMC) | <http://www.internal-displacement.org/> |
| International Consortium for Emergency Contraception | <http://www.cecinfo.org/> |
| International Development Research Centre | <https://www.idrc.ca/en> |
| International Federation of Red Cross and Red Crescent Societies | <https://media.ifrc.org/ifrc/> |
| International Medical Corps | <https://internationalmedicalcorps.org/> |
| International Organization for Migration | <https://www.iom.int/> |
| International Planned Parenthood Federation | <https://www.ippf.org/> |
| International Rescue Committee | <https://www.rescue.org/> |
| Ipas | <http://www.ipas.org/> |
| Jhpiego | <https://www.jhpiego.org/> |
| JICA | <https://www.jica.go.jp/english/> |
| John Snow International, Inc. | <https://www.jsi.com/JSIInternet/> |
| Marie Stopes International | <https://mariestopes.org/> |
| Maternity Foundation | <https://www.maternity.dk/> |
| Measure Evaluation | <https://www.measureevaluation.org/measure> |
| Medecins du Monde | <https://www.medecinsdumonde.org/en> |
| Medecins Sans Frontieres | <http://www.msf.org/en> |
| Mercy Corps | <https://www.mercycorps.org/> |
| Norwegian Agency for Development Cooperation | <https://www.norad.no/en/front/> |
| Norwegian Church Aid | <https://www.kirkensnodhjelp.no/en/> |
| Norwegian Refugee Council | <https://www.nrc.no/> |
| Oxfam | <https://www.oxfam.org/> |
| Pathfinder International | <http://www.pathfinder.org/> |
| Plan International | <https://plan-international.org/> |
| Population Council | <https://www.popcouncil.org/> |
| RAISE Initiative, Columbia University | <https://www.mailman.columbia.edu/research/reproductive-health-access-information-and-services-emergencies> |
| Refugee Law Project | <https://www.refugeelawproject.org/> |
| Refugee Rights Europe- formerly Refugee Rights Data Project (RRDP) | <http://refugeerights.org.uk/> |
| Save the Children | <https://www.savethechildren.org/> |
| Sexual Rights Initiative | <http://www.sexualrightsinitiative.com/> |
| Sexual Violence Research Initiative | <http://www.svri.org/> |
| Spanish Agency for International Development (AECID) | <http://www.aecid.es/EN/aecid> |
| Terre Des Hommes | [https://www.terredeshommes.org/#](https://www.terredeshommes.org/) |
| UN OCHA | <https://www.unocha.org/> |
| UN Women | <http://www.unwomen.org/en> |
| UNFPA | <https://www.unfpa.org/> |
| UNHCR | <http://www.unhcr.org/> |
| UNICEF | <https://www.unicef.org/> |
| UNISDR | <https://www.unisdr.org/> |
| USAID | <https://www.usaid.gov/> |
| What Works to Stop Violence | <http://www.whatworks.co.za/> |
| Women Deliver | <http://womendeliver.org/our-work/> |
| Women's Refugee Commission | <https://www.womensrefugeecommission.org/> |
| World Health Organization | <http://www.who.int/> |
| World Vision | <https://www.worldvision.org/> |
